# Modelling 30-day hospital readmission after discharge for COPD patients based on electronic health records

**DOI:** 10.1038/s41533-023-00339-6

**Published:** 2023-04-10

**Authors:** Meng Li, Kun Cheng, Keisun Ku, Junlei Li, Hao Hu, Carolina Oi Lam Ung

**Affiliations:** 1 State Key Laboratory of Quality Research in Chinese Medicine, Institute of Chinese Medical Sciences, University of Macau, Macao SAR, China; 2 School of Public Health, Southeast University, Nanjing, China; 3 Internal Medicine Department, Kiang Wu Hospital, Macao SAR, China; 4 Department of Public Health and Medicinal Administration, Faculty of Health Sciences, University of Macau, Macao SAR, China

**Keywords:** Health services, Outcomes research

## Abstract

Chronic Obstructive Pulmonary Disease (COPD) is the third most common chronic disease in China with frequent exacerbations, resulting in increased hospitalization and readmission rate. COPD readmission within 30 days after discharge is an important indicator of care transitions, patient’s quality of life and disease management. Identifying risk factors and improving 30-day readmission prediction help inform appropriate interventions, reducing readmissions and financial burden. This study aimed to develop a 30-day readmission prediction model using decision tree by learning from the data extracted from the electronic health record of COPD patients in Macao. Health records data of COPD inpatients from Kiang Wu Hospital, Macao, from January 1, 2018, to December 31, 2019 were reviewed and analyzed. A total of 782 hospitalizations for AECOPD were enrolled, where the 30-day readmission rate was 26.5% (207). A balanced dataset was randomly generated, where male accounted for 69.1% and mean age was 80.73 years old. Age, length of stay, history of tobacco smoking, hemoglobin, systemic steroids use, antibiotics use and number of hospital admission due to COPD in last 12 months were found to be significant risk factors for 30-day readmission of CODP patients (*P* < 0.01). A data-driven decision tree-based modelling approach with Bayesian hyperparameter optimization was developed. The mean precision-recall and AUC value for the classifier were 73.85, 73.7 and 0.7506, showing a satisfying prediction performance. The number of hospital admission due to AECOPD in last 12 months, smoke status and patients’ age were the top factors for 30-day readmission in Macao population.

## Introduction

Chronic obstructive pulmonary disease (COPD) is a major chronic disease characterized by slowly growing airflow obstruction^1^, which was projected to be the third leading cause of death worldwide by 2030^2^. From 1990 to 2015, the global prevalence of COPD increased by 44.2%^3^. It is estimated that 328 million people have COPD worldwide^4^. In particular, there are approximately 99.9 million COPD patients in China, and more than 900,000 people die prematurely from COPD each year^5^. The incidence of COPD among the younger population (people aged 40 years old and over) estimated to be 13.7% is also increasing, posing substantial economic and social burden on both patients and healthcare systems^6^. COPD is often accompanied by exacerbations of respiratory symptoms requiring admission to the hospital, where the cost of hospitalizations accounts for 75% of the total direct healthcare cost for COPD^7^. Acute exacerbations of COPD (AECOPD) is defined as a sustained worsening of patient’s symptoms from stable states, which is the most common cause of COPD-related hospitalizations^8^.

As the condition of COPD deteriorates, many COPD patients experience loss of function and are subject to high risk of admitting to the hospital repeatedly^9^. Readmission is usually measured by a 30-day readmission (hospital revisits within 30 days after discharge), which has been continuously rising worldwide in the last decade. COPD is one of the diseases with the highest rate of readmission within 30 days, along with congestive heart failure and pneumonia^10^. One research in UK showed that nearly 1 in 5 patients with COPD exacerbations had readmission at least once within 30 days after discharge. Reducing readmission is one of the priorities for some health systems, as hospital will otherwise be imposed financial penalties. The U.S. Centers for Medicare and Medicaid Services included COPD into its Hospital Readmission Reduction Program in 2014, which applied additional fines to the hospital when the readmission rate of medical insurance patients is too high^11,12^.

Considering the risk factors for COPD readmission remain largely unknown, more and more studies have predicted the risk of 30-day readmissions and developed predictive tools in recent years. Conventional methods based human experience may be paradigm to some extent^13^. However, these methods are also subject to a few limitations. For example, the experience may vary among physicians depending on different clinical backgrounds and experiences and may result in inconsistent decisions for the same case. The human experience is not easily transferable or adaptive when the characteristics of patients changes, which is particularly the case in qualitative experience^14^. Recently, the advantages of machine learning methods in predicting the prognosis of patients have received much attention, the key of which is to deal with a complex nonlinear relationship between predictor variables and outcome indicators to produce more reliable predictions^15–17^. The machine models are built in an automatic manner via labelled dataset and so are quantitative, transferable and adaptive. Despite these advantages, there are only a few studies on their ability to predict 30-days readmission after discharging due to COPD, especially in Asian countries and regions. In addition, different machine learning models have different levels of model interpretability, which is also of high interest in helping practitioners/clinicians understand how a specific decision is made in machine learning models. However, the studies focused on interpretable machine learning models (e.g., decision tree), which are closer to human decision-making processes is even rarer.

The aim of this study is to develop a predictive decision tree model based on the data from the electronic health record (EHR) system to define and predict the risk factors that affect the 30-day readmission of COPD patients in Macao. The findings would be beneficial to formulating specific suggestions that manage the controllable risk factors to prevent readmission, and therefore avoid the negative impact of patients’ prognosis and reduce their medical costs and disease burden.

## Results

### Descriptive statistics

A total of 782 hospitalizations for AECOPD were enrolled in this study. Among them, 207 (26.5%) were selected as derivation sample due to readmission after the discharge of 30 days. Then 207 records were randomly selected from the remaining data (73.5%) that were not re-admitted to the hospital. As a result, a balanced dataset including 207 records with readmission and 207 records without readmission was generated. Descriptive information for the balanced dataset was shown in Table 1. It followed from Table 2 (end of this document) that male accounted for 69.1%. The mean age is 80.73 years old, 14.25% and 49.28% of patients were current or previous smokers, only 11.59% of patients did not have any comorbidities, mean LOS in this study is 12.99 days. For the blood test results, 71.74% of patients were with blood eosinophils < 300 cells/μL. 34.06%, 56.28%, 8.70% of patients’ Hemoglobin, WBC and Creatinine were within normal range. Regarding to clinical therapies, 47.58% and 76.57% used systemic steroids and antibiotics, respectively. 89.13% received oxygen therapy, and 85.99% did not receive NIV. Only 14.73% had pulmonary rehabilitation during hospitalization.

**Table 1:** Inclusion variables included three categories in this study.

Patients’ information	age, gender, history of tobacco smoking, number of comorbidities (NOC), length of stay (LOS), number of hospital admission due to COPD in last 12 months (NoH-12)
Blood tests	BEC, hemoglobin, WBC and creatinine
Clinical therapies	systemic steroids (prednisolone, dexamethasone, methylprednisolone) and antibiotics, oxygen therapy, NIV and PR and inhaled medications

**Table 2:** Demographic information.

Variables	*n* (%)	Variables	*n* (%)
**Gender**		**WBC (10^9/L)**	
Male	286 (69.0%)	Below normal range	6 (1.5%)
Female	128 (31.0%)	Normal range	233 (56.3%)
**Age (years)**		Above normal range	166 (40.1%)
Median (IQR)	82 (73-88)	Missing	9 (2.2%)
Mean (SD)	80.73 (10.1)	**Creatinine (μmol/L)**	
**History of tobacco smoking**		Below normal range	269 (65.0%)
No	148 (35.7%)	Normal range	36 (8.7%)
Yes	59 (14.3%)	Above normal range	82 (19.8%)
Quit	204 (49.3%)	Missing	27 (6.5%)
Missing	3 (0.7%)	**Systemic steroids**	
**NoC**		No	215 (51.9%)
0	48 (11.6%)	Yes	197 (47.6%)
1	102 (24.6%)	Missing	2 (0.5%)
2	117 (28.2%)	**Oxygen therapy**	
3	99 (23.9%)	No	42 (10.2%)
4 or more	48 (11.6%)	Yes	369 (89.1%)
**LOS**		Missing	3 (0.7%)
Median (IQR)	11.00 (6.0–17.0)	**Noninvasive ventilation**	
Mean (SD)	12.99 (8.6)	No	356 (86.0%)
**NoH-12**		Yes	56 (13.5%)
No	127 (30.6%)	Missing	2 (0.5%)
1–3 times	153 (37.0%)	**Pulmonary rehabilitation**	
More than 3 times	132 (31.9%)	No	351 (84.8%)
Missing	2 (0.5%)	Yes	61 (14.7%)
**BEC (cells/uL)**		Missing	2 (0.5%)
Below 300 cells/uL	297 (71.7%)	**30 days readmission**	
Above 300 cells/uL	108 (26.1%)	No	207 (50.0%)
Missing	9 (2.2%)	Yes	207 (50.0%)
**Hemoglobin (g/L)**		**Inhaled medications**	
Below normal range	254 (61.3%)	Group 1	74 (17.9%)
Normal range	141 (34.1%)	Group 2	79 (19.1%)
Above normal range	9 (2.2%)	Group 3	234 (56.5%)
Missing	10 (2.4%)	Group 4	27 (6.5%)
**Antibiotics**			
No	97 (23.4%)		
Yes	317 (76.6%)		

Note: BEC was put into “Below” and “Above” categories by using a threshold of 300 cells/μL; Hemoglobin, WBC was put into “Below”, “Normal” and “Above” categories by using <4, 4–10 and >10, respectively. Creatinine was put into “Below”, “Normal” and “Above” categories by considering gender difference (normal range for male: 53-106 μmol/L; normal range for female: 44–97 μmol/L). NoC was categorized into categorical variable [0,1,2,3,4+], where 4+ means there was 4 or 4+ comorbidities. While NoH-12 was transformed into three categories including [0, (1,2,3), (4,5,5+)].

### Feature selection

The quantitative results of KS test for continuous variable selection were also summarized in Table 3, where the third row “logical” indicates whether the null hypothesis was rejected by comparing the second-row “*p* value” against the significant level at $\alpha = 0.05$. It showed that both

**Table 3:** Result of KS test for continuous variable.

	Age	LOS
**P-value**	3.52 e^-05^	4.82 e^-04^
**Logical**	1	1

Age and LOS were significant for readmission modelling.

The results of Chi-Square test for categorical variables were summarized in Table 4. It follows that the significant features (highlighted in bold) included Smoke, Hemoglobin, Steroid, Antibiotics and NoH-12. As a result, there are a total of 7 significant features being selected for the decision tree classifier construction, which include continuous variables (Age, LOS) and categorical variables (Smoke, Hemoglobin, Steroid, Antibiotics, NoH-12).

**Table 4:** Results of Chi-Square test for categorical variable.

	Gender	Smoke	NoC	BEC	Hemoglobin	WBC	Creatinine
P-value	0.20	**6.15e**^**-06**^	0.15	0.93	**8**.**24e**^**-04**^	0.68	0.30
	**Steroid**	**Antibiotics**	OT	NIV	PR	**NoH-12**	Inhaled Medications
P-value	**0**.**023**	**0**.**028**	0.19	0.77	0.21	**4**.**93e**^**-16**^	0.470

### Preliminary classifier comparisons

The results of the preliminary classifier performance comparison were summarized in this part. In particular, there is a classifier App available in MATLAB, entitled “classificationLearner”, where 22 typical classification models, including Decision trees, Logistic Regression (LR), Naïve Bayes, Support Vector Machines (SVM), Ensemble approaches, Neural Networks, and Kernel approaches can be quickly tested in term of accuracy. The comparative results in terms of accuracy via five-fold cross-validation were summarized in Table 5.

**Table 5:** Comparation results of 22 classification models in term of accuracy via five-fold cross-validation in MATLAB “classificationLearner”.

Decision trees			Logistic regression	Naïve Bayes		SVM	
Fine	Medium	Coarse	LR	Gaussian	Kernel	Linear	Quadratic
63.5%	69.1%	72.2%	66.7%	66.9%	68.1%	67.9%	63.8%
**Support Vector Machines (SVM)**				**Ensemble approaches**			
Cubic	Fine Gaussian	Medium Gaussian	Coarse Gaussian	Boosted	Bagged	RUS Boosted	
62.3%	63.3%	67.4%	65.7%	69.3%	65.0%	69.1%	
**Neural Networks**					**Kernel**		
Narrow	Medium	Wide	Bi-layered	Tri-layered	SVM	LR	
60.6%	58.5%	59.4%	60.9%	60.6%	56.0%	57.7%	

It followed from Table 5 that the decision tree model (un-optimized) possessed the best performance in term of accuracy and therefore was selected for further study in this work.

### Decision tree classifier

#### Hyperparameter optimization

**Fig. 1:**
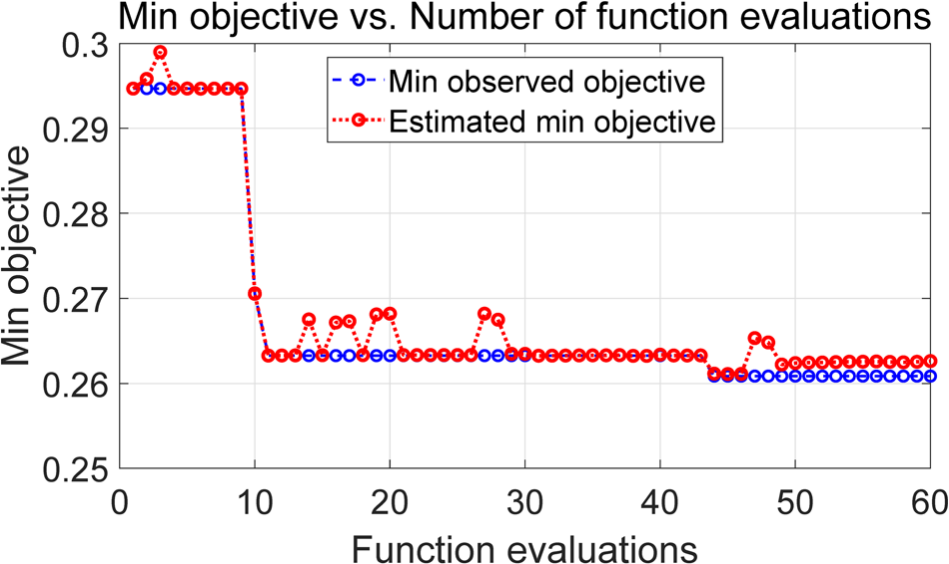
Bayesian optimization loss of decision tree over function evaluations. Blue line represents the minimum observed objective and red line represents the estimated minimum objective.

The results of decision tree modelling were then summarized. To automatically tune the hyperparameters of the decision tree model by Bayesian optimization, three key hyperparameters were considered, including MinLeafSize ([1, max(2, floor(NumObservations/2))]), MaxNumSplits ([1, max(2,NumObservations-1)]) and SplitCriterion (e.g. gdi, deviance). The maximum objective evaluations were chosen as 60. The objective functions against the function evaluations (i.e., iteration) were displayed in Fig. 1. By using the Bayesian hyperparameter optimization, the classification loss of decision tree was decreased from 29.2% to 26.1%.

#### Confusion matrix

**Fig. 2:**
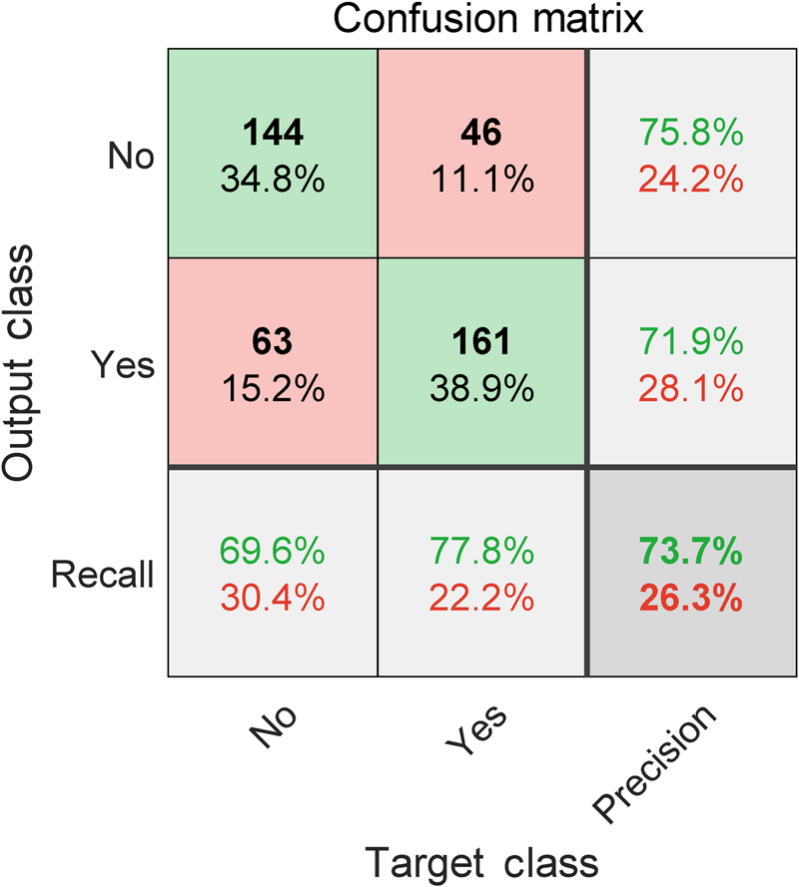
Confusion matrix of the optimized decision tree model. Blue diagonal and red non-diagonal elements represent the correct and wrong prediction, respectively.

The confusion matrix of the decision tree optimized by Bayesian approach was displayed in Fig. 2. The target and output class represent the ground truth class and the predicted class, respectively. The diagonal cells in green display the number/percentage of the correct classification, while the off-diagonal cells are where the misclassification happens. For No class (without readmission), 144 in green is TP and 46 in red is FP, 63 in red is FN and 161 in green is TN. So precision for No class is 144/ (144+ 46) = 75.8%, while recall for No class is 144/(144+63) = 69.6%. Similarly, precision and recall for Yes class are 71.9% and 77.8%. As a result, the mean precision and recall for the decision tree classifier are 73.9% and 73.7%. The cell at the bottom right displays the overall accuracy (73.7%). These metrics for the balanced training dataset implied an acceptable performance.

#### ROC and AUC

**Fig. 3:**
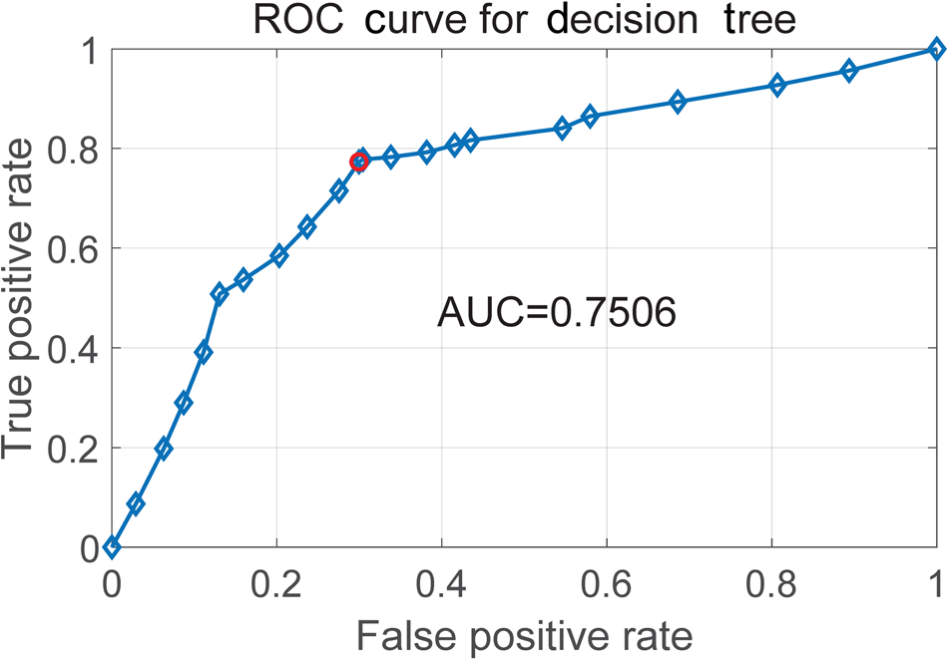
ROC curve of the optimized decision tree. The blue line represents the pair of true positive rate against false positive rate and AUC value denotes the area under the ROC curve.

The ROC curve of the optimized decision tree was displayed in Fig. 3 with an AUC value of 0.7506, which showed the diagnostic ability of the binary classifier system as its discrimination threshold was varied. An AUC value of 0.7506 meant the developed model was considered to be acceptable.

#### Predictor importance

The predictor importance values returned by the optimized decision tree classifier could be obtained, where the most important variable in predicting readmission was the No of hospitalizations in the past 12 months, followed by smoking status and then age. While other variables, such as length of hospitalization stay, hemoglobin, steroid, antibiotics, although being selected in feature selection stage by using KS test (continuous variables) or Chi-Squared test (categorical variables), had very low importance estimates returned by the optimized decision tree.

#### Decision rules

**Fig. 4:**
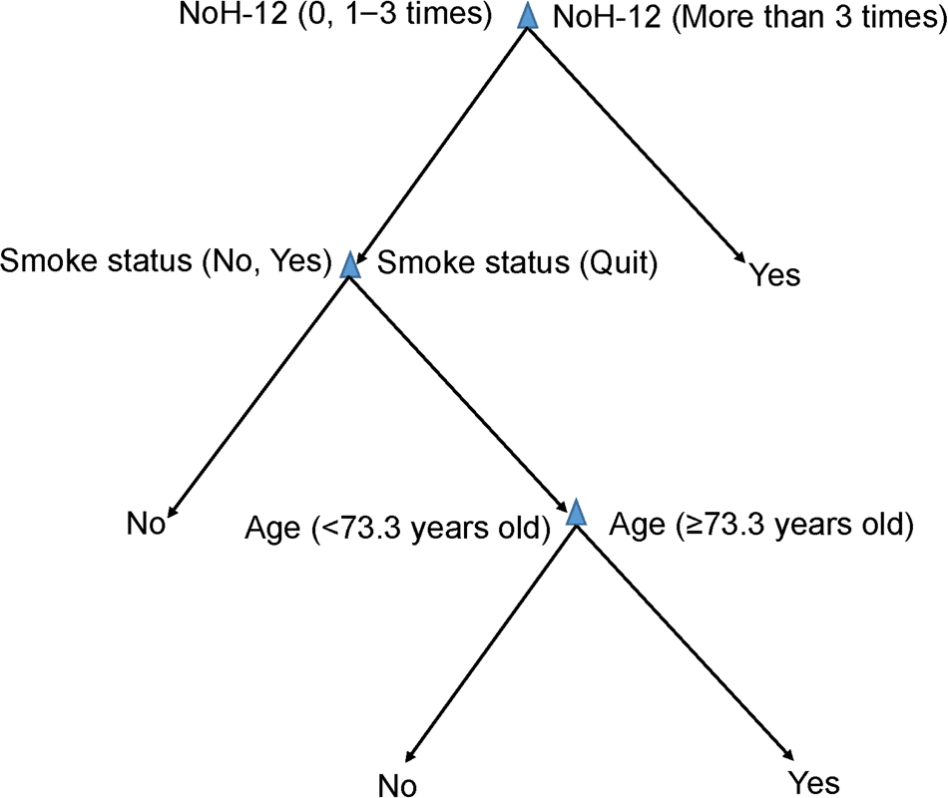
Visualization of the decision tree in a flowchart-like structure. The decision rule has a hierarchical, tree structure consisting of a root node, branches, internal nodes and leaf nodes.

The optimized decision tree was also displayed in Fig. 4 in a flowchart-like structure (a set of if-else conditions), where the paths from root to leaf represented the classification/prediction rules. In this figure, each node represented a test on a feature, each branch represented the outcome of the test, and each leaf (or terminal) node represented a class label. For example, the categorical variable NoH-12 was the root node, which was splitted into two branches. If its value fell into categorical 2 (more than 3 times), then the prediction was Yes, while if its value fell into categorical 0 and 1 (1–3 times), then the decision would go to smoke node for further test. It is believed that these if-else conditions in the decision tree model are easily understandable to human beings.

## Discussion

In this study, the readmission rate of COPD patients within 30 days of discharge was found to be 26.5%. A data-driven decision tree-based modelling approach with Bayesian hyperparameter optimization was developed. The key predictors of readmission included patients’ comorbidities, the length of stay during previous admission, and the number of previous admissions. The mean precision-recall and AUC value for the decision tree classifier were 73.85%, 73.7% and 0.7506, showing a satisfying prediction performance. The similarities and differences of this study over the existing ones will be discussed in terms of readmission rate, contributing factors, and the model performance will be discussed further in the following.

The readmission rate of patients who readmitted to the hospital within 30 days of discharge from hospitalization due to COPD was 26.5%, which was slightly higher that the readmission rate found in another studies^17–21^. One study conducted in an Australian tertiary hospital found that the 30-days hospital readmission rate was 25%^19^. There are also serval studies focused on the 30-days readmission of COPD patients in the US. One research found that during 2003-2004, 22.6% of fee-for-service Medicare beneficiaries admitted to the hospital for COPD were readmitted within 30 days^20^. The readmission rates in similar studies in 2015 and 2019 were 20.2% and 21%, respectively^21^. In the United Kingdom, approximately a quarter (24%) of patients with COPD exacerbations were readmitted at least once within 30 days of discharge^22^. Researchers in London found that the 30-days readmission rate of AECOPD patients in London is between 14%-20%^23^. The higher readmission rate in this study may be partly explained by the age of the patients included in the studies. The patients in this study generally had a higher average age (80.73 years old) and most of them were combined with more than 1 type comorbidity (88.3%), indicating that the patients in this study might be subject to more serious health conditions contributing to a higher readmission rate.

Regarding the predictors of readmission after hospitalization, comorbidities, length of stay and previous admissions were frequently cited as predictors for readmission^24^. Regarding COPD-specific predictors analysis, Sharif et al found that history of heart failure, lung cancer, anxiety, depression, osteoporosis, and length of hospital stay were associated with higher likelihood of readmission within 30 days^25^. Poor lung functions like lower oxygen tension and dyspnea were identified as risk factors to COPD readmission^26^. COPD patients with acute hypercapnic respiratory failure, who had treatment with noninvasive ventilation have a higher risk of readmission and life-threatening events^27^. One study showed that weight loss during hospitalization and low body mass index were associated with unplanned readmission^28^. In addition to predictors related to high risk of readmission, a research in US found that any level of moderate or vigorous physical activity had a significantly lower risk of 30-day readmission compared with inactive patients by conducting multivariate adjusted analysis^29^. The use of nutritional supplementation for COPD inpatients was related to reduced 30 days readmission rate^30^. The risk factors affecting 30-day readmission of COPD patients in this study included NoH-12, smoking status, and patient’s age, which were basically consistent with previous studies. Frequency of NoH-12 is closely linked to frequent COPD exacerbations, which requires hospital admission and is combined with a higher mortality^31^. The use of inhaled medications should be able to reduce exacerbations due to their benefits in reducing bronchospasm and inflammation, and relieving ongoing breathing problems^32,33^. On the other hand, it has also been shown that the use of inhaled medications were positively associated with a longer length of stay during hospitalization^34^, since the use of different inhaled medications was possibly associated with COPD severity as well. Nevertheless, the results in this study indicate that the use of different inhaled medications was not a significant factor for COPD readmission with 30 days after discharge. Further investigation is needed to explore the patients’ compliance and the accuracy of the techniques when using the inhaled medications as the proper use of inhaled medications as prescribed have been shown to have an impact on both COPD exacerbations and COPD severity, which would in turn have effects on COPD readmission.

There was one significant inconsistency with other study findings in this study. Current smokers would not readmit to hospital within 30 days after discharge, which could be explained by the lower average age of current smoking group after in-depth investigation (current smoker: 74.83 years old, quitting smoking: 80.29 years old, non-smoker: 83.70 years old). This finding implied that the current smokers in our study might have a milder condition, because the number of comorbidities and risk of exacerbation increased with age^35^. Smoking had been proven to be the primary risk factor for COPD, causing irreversible damage to the lungs, so even patients who quit smoking still have a worse condition than patients who do not smoke^36^. Smoking cessation at an early stage of COPD should be taken as a priority when trying to improve COPD prognosis^37^.

Based on previous studies, the LACE index is a common model used to assess the risk of a patient’s 30-day readmission or death. The parameters include: Length of stay, acuity of admission, co-morbidities, and emergency department visits within the last 6 months^19^. One research applied LACE index to COPD patients from 11 hospitals of Ontario during 2002-2004, where an AUC value of 0.684 was generated by the model^38^. Nevertheless, the results from another research in Australian shown that LACE index had moderate discriminative ability to predict 30-day readmission (AUC=0.63)^19^. Bashir et al found LACE index was not associated with readmission, and universal prediction model for readmission might not be achievable^39^.

In this study, the overall accuracy is 73.7%, and AUC value of 0.7506. Although they are relatively high and acceptable, direct comparison of different predictive models is futile because the data-driven models will be changed according to data included, and the selection of parameters is also mutative. The advantages of this study can be summarized in the following aspects. Firstly, compared with traditional decision-making process conducted by physicians, machine learning methods are more consistent specific. Secondly, this research adopts a decision tree model with if-else conditions (or rules), which is easy to understand and interpret since it is like human decision-making process. Thirdly, the proposed framework integrating feature selection, decision tree classifier and Bayesian hyperparameter optimization is applicable to different classification problems in public health. This system could learn and self-improve and therefore more precise results will be recalculated when more new data is available. Finally, this is the first study on the readmission of COPD population in Macau with high-quality dataset, since the patient data is centralized, and rarely no patients were referred to another hospital for readmission.

Some limitations exist in this study mainly in terms of training data and modelling methods. In term of training data for model construction, most pulmonary function test data was either incomplete or unavailable in the EHR to allow categorization of the COPD patients according to the GOLD (Global Initiative for Chronic Obstructive Lung Disease) guideline. However, due to the included patients were all admitted for acute exacerbation of COPD, they were considered either Grade C or Grade D by the specialists; 2) we had a limited number of independent variables (features), and more clinical indicators including lung functions may provide a more accurate COPD readmission prediction; 3) at the same time, the sample size (e.g., number of observations) is relatively small, relevant studies will be carried out in the future to enlarge the training data for a more reliable prediction model; in addition, the prospective validation method, instead of the 5-fold cross-validation in the current study, can also be considered with the future advent of a relatively large labelled dataset. In term of modelling method in this study, 1) the current data-driven analysis and modelling approach could not present the causal relationship, which means the results may change by using various dataset (e.g., data with different characteristics); 2) although the decision tree model in this study is simple and relatively transparent, may not be able to be modelling very complex relationships between features and response variable. As a result, with the advent of a large amount of training dataset in the future, a more reliable (e.g., stable, accurate) modelling will be investigated by using more complex modelling methods (e.g., random forest).

Predictive models of readmission after discharge may serve as a tool that assists clinicians in developing treatment strategies specifically targeting those at a high risk of hospitalization and readmissions. A data-driven decision tree-based modelling approach with Bayesian hyperparameter optimization was developed for identifying discharged COPD patients with high risks of being readmitted within 30 days based on the health records of COPD inpatients from the EHR system of Kiang Wu Hospital, Macao. More clinical and lung conditions data are needed to expand the implications of this research. A set of if-else conditions were generated by the decision tree model with an overall accuracy of 73.7%, and an AUC of 0.7506. Moreover, the predictor importance values returned by the optimized decision tree classifier showed that the top factor for the readmission was the number of hospital admission due to AECOPD in last 12 months, followed by smoke status and patients’ age. Reducing readmission rate could lead to less administrative burden and benefit to reduce patients’ economic burden and quality of life. It is necessary for COPD patients to start smoking cessation in an early stage to reduce potential risks of readmission and related disease burden.

## Methods

### Data collection

Obstructive airway disease is one of the ten leading causes of death in Macao. Studies have shown that second-hand smoke affects 14% of the local labor force, increasing the incidence and mortality of COPD in Macao. Kiang Wu is one of the three major hospitals in Macao, which accounts for 47% of total resources. In this study, we reviewed the health records of COPD inpatients from the EHR system of Kiang Wu Hospital from January 1, 2018, to December 31, 2019. The criteria of inclusion were: (1) patients admitted with a main diagnosis of COPD (International Classification of Diseases-10 codes (ICD-10): J44); and (2) admission due to acute exacerbation as confirmed by the specialists. It is noted that the labeled data and also the trained prediction model in the study is site-specific for regions with similar patient characteristics, although the overall methodology is transferable to other regions or studies.

### Variables and measurements

There were 3 categories of data in this study including demographic data, blood test results and clinical therapies (See Table 1). Patients’ demographic data included age, gender, history of tobacco smoking, number of comorbidities (NoC) and number of hospitalizations in the past 12 months (NoH-12). Blood test results included blood eosinophil count (BEC), hemoglobin, white blood cells (WBC) and creatinine. Clinical therapies for COPD in Macao included data about the usage of systemic steroids (prednisolone, dexamethasone, methylprednisolone) and antibiotics, oxygen therapy, noninvasive ventilation (NIV) and pulmonary rehabilitation (PR). It is noted that there are too many combinations of inhaled medications and so it would be inappropriate to directly treat it as a categorical variable considering the limited number of samples in this study. Therefore, we divide the inhaled medications into a few categories. Following the work^34^, according to the use of inhaled medications of the COPD, the hospitalization records were assigned to 1 of the 4 groups. Group 1 included the records who used only one type of inhaled medication (e.g., “LABA, LAMA or both”, “SABA, SAMA or both” or ICS only.). Group 2 included the records who received two types of inhaled medications (e.g., “(LABA, LAMA or both) and (SABA, SAMA or both)”, “(LABA, LAMA or both) and ICS” or “(SABA, SAMA or both) and ICS”.). Group 3 included the records who used the combination of all 3 types of inhaled medications (e.g., “(LABA, LAMA or both) and (SABA, SAMA or both) and ICS”). Group 4 referred to the records in which the patients did not use any inhaled medications. It is noted that some variables such as BEC, hemoglobin, WBC, creatinine are indeed varied at every admission for an individual patient due to illness or drug effects, and therefore, this study used data on hospitalization information per patient admission to reflect the dynamic readmission risk.

### Data discretization and balancing

Some continuous variables (e.g., BEC, hemoglobin, WBC, creatinine, NoC, NoH-12) were first transformed into categorical variables based on their proper reference ranges. Data imbalance problem, the distribution of examples across different classes is biased or skewed, generally poses a challenge for predictive modelling that the predictive performance is usually poor, specifically for the minority classes (the ones with fewer samples). Considering that in this study the numbers of records with readmission (Yes class) and without readmission (No class) were significantly different, data balancing technique was conducted to generate a balanced dataset for data-driven classification model construction. In particular, down-sampling approach was adopted in this study. In this approach, all records of Yes class (the one with fewer samples) were first preserved, then random sampling was performed by using “randperm” function (i.e., random permutation of integers) in MATLAB R2020b for the records of No class so that the number of randomly sampled records of No class had the same size as the Yes class. Therefore, a balanced dataset consisting of the same number of records for Yes and No classes was generated for the following data analysis and classification model construction.

### Data analysis and feature selection

#### Descriptive analysis

Descriptive analysis was first performed for the continuous variables and categorical variables in the balanced dataset. In particular, for continuous variables, mean and median were adopted, while for categorical variables the number and proportion for different classes were summarized.

#### Feature selection

Considering that there was 15+ features (i.e., candidate independent variables) and a limited number of available samples (i.e., No. of records) for model construction, feature selection was performed to remove the irrelevant and redundant features so that a simpler and more reliable model can be derived for prediction. The feature selection methods for continuous and categorical variables were introduced below.

For continuous variables, in order to assess their distribution differences under Yes and No classes, the two-sample Kolmogorov-Smirnov test (KS test) was adopted. KS test is a general nonparametric statistical approach to quantify whether two samples come from the same distribution or not. Suppose two samples of size m and n with the observed/empirical cumulative distribution functions F(x) and G(x), the KS statistic is defined by

1 $${{{\mathrm{D}}}}_{{{{\mathrm{m}}}},{{{\mathrm{n}}}}} = \sup _x|F_m(x) - G_n(x)|$$

where sup is the supremum function. The null hypothesis is that the samples are drawn from the same distribution, and one rejects the null hypothesis (at a significant level α) if D_m,n_ > D_m,n,α_ where D_m,n,α_ is the so-called critical value. For sufficient large m and n,

2 $${{{\mathrm{D}}}}_{{{{\mathrm{m}}}},{{{\mathrm{n}}}},\alpha } = {{{\mathrm{c}}}}\left( \alpha \right)\sqrt {\frac{{m + n}}{{mn}}}$$

where c(α) is the inverse of the Kolmogorov distribution at α, given by ${{{\mathrm{c}}}}\left( \alpha \right) = \sqrt { - 0.5 \ast \ln \left( {\alpha /2} \right)}$. In this study, the “kstest2” function in MATLAB R2020b was adopted with α = 0.05.

For categorical variables, Chi-Square test of independence was adopted. Chi-Square test is a statistical hypothesis test that assumes (the null hypothesis) the observed frequencies for a categorical variable match the expected frequencies for the categorical variable, i.e., H_0: “variable 1 is independent of variable 2”. Therefore, it is usually used to determine whether there is an association between two categorical variables or not. In this study, Chi-Square statistics (along with its p-value) between the candidate categorical variables and the dependent variable (readmission or not) were returned by using the “crosstab” function (e.g., cross-tabulation) in MATLAB R2020b.

### Classification model

#### Decision tree model

Upon choosing the features, the next step is to build a classification model by using machine learning approaches. Different machine learning-based classification models are available in literature such as classification tree, logistic regression, Naïve Bayes, Support Vector Machines (SVM), Ensemble approaches, Neural Network among others. Different models have their own pros and cons in terms of accuracy, computation load, transparency, interpretability and reliance on a large labelled dataset. In this study, upon a preliminary performance comparison in term of accuracy via five-fold cross-validation in MATLAB App “classificationLearner”, decision tree model, a so-called white box model (against black-box or grey box models), is adopted. In particular, the main rationale for choosing the decision tree model are also summarized as below. First, in the preliminary performance comparison, decision tree-based approach possesses the best performance in term of accuracy. Second, decision tree is simple to understand and interpret since its inherent transparency and interpretability can help users follow the path of the tree and therefore understand the decision rules (i.e., if-else rules). Third, the simplicity of the model also makes it have a less reliance on a large training dataset compared against complex models such as neural network models. Fourth, predictor importance values can also be estimated in the decision tree, which can be used to assess the importance of different variables in making the decision. It is also noted that the missing data problem in the training dataset can be automatically handled by the decision tree model (e.g., “fitctree” in MATLAB environment).

Like many other machine learning models, there are hyperparameters in decision tree algorithm which have effects on its performance and should be properly tuned. The hyperparameters include the ones controlling the tree depth (e.g., MaxNumSplits, MinLeafSize or MinParentSize) and Split Criterion (e.g., gdi, deviance). Different approaches (e.g., grid search, random search, Bayesian optimization) are available to systematically tune these hyperparameters in order to get satisfying performance; in this study Bayesian parameter optimization (a sequential model-based optimization) was adopted due to its promising performance (efficiency) in deriving a good solution in a limited amount of steps/time. In addition, 5-fold cross-validation (against hold-out validation) was adopted to maximally use the limited amount of dataset, gain stable predictions and also avoid the problem of overfitting (i.e., gaining good performance on the training dataset but poor performance on testing dataset). The decision tree algorithm with Bayesian hyperparameter optimization is summarized in supplementary martials.

#### Performance evaluation

Metrics to evaluate the performance of machine learning classification models are also introduced in this part. True Positive (TP) denotes the correctly predicted positive values; False Positive (FP) is the scenario where the actual class is negative, but the predicted class is positive; and False Negative (FN) represents the scenario that the actual class is positive, but the predicted class is negative. From these definitions, different metrics can then be defined for performance evaluation. For instance, Accuracy is a good measure for symmetric datasets (i.e., the number of each class has the same order of magnitude). Precision and Recall are also commonly used for performance evaluation, particularly for data with uneven class distribution. These values are usually first calculated for each class, and their mean values for different classes are then chosen. Accuracy, Precision and Recall for a specific class are defined by formula below (3), which can be calculated by using confusion matrix.

3 $$Accuracy = \frac{{{\sum} {TP} }}{{ALL}},\;{\it{Pr}}ecision = \frac{{TP}}{{TP + FP}},\;{\it{Re}}call = \frac{{TP}}{{TP + FN}}$$

A receiver operating characteristic curve (also termed ROC curve) is a graphical plot illustrating the classification ability of a binary classifier, where the true positive rate against the false negative rate is plotted at various thresholds (for classification). Upon plotting ROC, area under the ROC curve (AUC) is an effective manner to summarize the overall accuracy, which takes value from 0 to 1. In general, an AUC of 0.5 suggests no discrimination, and 0.7 to 0.8 is considered acceptable, 0.8 to 0.9 is considered excellent, and more than 0.9 is considered outstanding^18^.

### Reporting summary

Further information on research design is available in the Nature Research Reporting Summary linked to this article.

## Supplementary information

Supplementary Material File

Reporting Summary

## Data Availability

The data that support the findings of this study are available on request from the corresponding author [COLU].
