# Supplementary material for: Modelling 30-day hospital readmission after discharge for COPD patients based on electronic health records
Source: NPJ Prim Care Respir Med. 2023 Apr 10;33:16. doi: 10.1038/s41533-023-00339-6 (PMC10086061; doi:10.1038/s41533-023-00339-6)
Supplement: Supplementary file 1 — Supplementary Material File [file 41533_2023_339_MOESM1_ESM.pdf]

Algorithm: Decision Tree with Bayesian hyperparameter optimization

(a) Parameter setting: Set certain stopping rules for the algorithm (e.g., evaluation number, 60 in this study) to end the iteration in Bayesian optimization;

(b) Optimization problem: Choose hyperparameters to be tuned (e.g. MinLeafSize, MaxNumSplits and SplitCriterion) as $\lambda \in \Omega$, then build the decision tree classification model with hyperparameters λ. Define the objective function as the 5-fold cross-validation loss and the optimization problem can be formulated as $\lambda opt = argmin\lambda \in \Omega Loss(\lambda)$.

(c) Bayesian optimization: Different from grid search or random search, Bayesian optimization is a sequential optimization algorithm, which suggests new parameters by sequentially doing: 1. fitting a Gaussian process model for data points, with new data point updating; 2. identifying new point for evaluation by maximizing the acquisition function.

(d) Optimized classifier: Stop evaluation when the stopping criterion is satisfied and return the optimized decision tree.
